# The Making of Evident Expertise: Transforming Chemical Analytical Methods into Judicial Evidence

**DOI:** 10.1007/s00048-021-00306-7

**Published:** 2021-08-02

**Authors:** Marcus B. Carrier

**Affiliations:** Fakultät für Geschichtswissenschaft, Philosophie und Theologie, Abteilung Geschichtswissenschaft, Universität Bielefeld, Postfach 10 01 31, 33501 Bielefeld, Germany

**Keywords:** Expertise, Forensic Toxicology, Comparison, Chemical Methods, Criminal Poisonings, Expertise, Forensische Toxikologie, Vergleich, Chemische Methoden, Vergiftungen

## Abstract

This article investigates the question of how forensic toxicologists established the credibility of chemical analytical methods in poisoning lawsuits in the nineteenth century. After encountering the problem of laypersons in court, forensic toxicologists attempted to find strategies to make their evidence compelling to an untrained audience. Three of these strategies are discussed here: redundancy, standard methods, and intuitive comprehensibility. Whereas redundancy was not very practical and legally prescribed standard methods were not very popular with most forensic toxicologists, intuitive comprehensibility proved effective and popular. This strategy relied on employing methods which did not require chemical knowledge to be understandable. The methods aimed to generate a visual aid and to be obvious in their results. Two forms of this strategy are discussed here: the presentation of the actual material and explicit comparison. I argue that this shift towards presenting forensic toxicology expertise as evident represents an important step in the history of forensic expertise.

## Abstract (de)

In diesem Artikel wird der Frage nachgegangen, wie im 19. Jahrhundert bei Vergiftungsfällen die Anwendung analytisch-chemischer Methoden Glaubwürdigkeit erlangte. Nachdem forensische Toxikologen Laien vor Gericht als ein ernstzunehmendes Problem identifiziert hatten, versuchten sie, Strategien der Anpassungen zu finden, um kritische Fragen vor Gericht zu vermeiden. Drei Strategien werden hier diskutiert: Redundanz, Normalmethoden und Anschaulichkeit. Während Redundanz an praktischen Problemen scheiterte und staatlich verordnete Normalmethoden bei den meisten forensischen Toxikologen unbeliebt blieben, hatte die Anschaulichkeit keines dieser Probleme. Die Idee dieser Strategie war es, Methoden zu nutzen, die kein chemisches Wissen voraussetzten, um nachvollziehbar zu sein. Sie dienten als eine Form der Visualisierung und führten zu auch für Laien recht offensichtlich wirkenden Schlussfolgerungen. Zwei Formen dieser Strategie werden hier vorgestellt: die Darstellung der eigentlich gesuchten Substanz und der explizite Vergleich. Ich werde argumentieren, dass diese Strategie, die eigene Expertise als evident zu präsentieren, einen wichtigen Schritt in der Geschichte der Forensik darstellte.

The poisoning or execution of a human by secretly administering a small dose of some substance, which is very dangerous in small quantities and easily concealed, is a deed so easy and in need of so little determination by the murderer that every vile and spiteful soul must be tempted to do this the more difficult it is to detect the culprit and to convict him for his evil action.^1^ (Müller 1800: 335)

This warning appears at the beginning of the physician Johann Valentin Müller’s (1756–1813) chapter on poisoning in his *Entwurf der gerichtlichen Arzneywissenschaft *(1800). His sentiment is clear: poisoning is easy because it does not require strength, it is executed quietly, and it is hard to detect. Poisons can be easily hidden in a meal, all that is required is a “vile soul.” Fears of poisoning were widespread during the nineteenth century (cf. Burney 2012: 11–19). However, it was also during this period that experts in analytic chemistry developed new methods focused on making the invisible act of poisoning visible. In this article, I will ask how expert scientists—in my case forensic toxicologists^2^—established their credibility and authority in courts regarding the usefulness of their analytic methods in poisoning cases. The literature on the history of forensic science has grown tremendously in recent years. There are now classic books on the emergence of legal medicine in both France (Chauvaud 2000) and the United States (Mohr 1993), as well as general textbooks on the history of (Western) forensic science (Watson 2011; Adam 2016). British toxicology has been analyzed by Ian Burney (2012) and the social history of poisoners and their victims in England and Wales was the focus of Katherine Watson’s work (2004). The articles edited by José Ramón Bertomeu-Sánchez and Agustí Nieto-Galan (2006) focus on the French toxicologist Mathieu Orfila (1787–1853) and his influence on toxicology as a discipline. Furthermore, whole books have been written about the wide dissemination of arsenic, which was the most commonly used poison during the nineteenth century (Whorton 2010; Parascandola 2012). The strong focus on Western, and especially British and French, but also U.S. American toxicology, has also been broadened to include more global perspectives in articles edited by Burney and Christopher Hamlin (2019). All these examples indicate a strong interest in the history of toxicology; yet little work has been done on the history of toxicology in Germany or the German states. Notable exceptions exist, of course. For example, the work of Bettina Wahrig (2006) on German toxicology textbooks in the late eighteenth and early nineteenth centuries, along with Julia Saatz’s (2018) dissertation on poisoning narratives come to mind. Meanwhile, Catherine Crawford (1994) used the inquisitorial trial in the Holy Roman Empire in a comparative case study to better understand the peculiarities in the early modern British adversarial system. In this article, however, the focus is on toxicological practice and its motivation. When German states implemented trial reforms around 1848, many forensic toxicologists feared they would suffer from a rhetorical disadvantage in public courts where their scientific expertise would be scrutinized and ultimately judged by laypeople, since judges, members of the jury, and lawyers lacked scientific training. I will argue that their analytic methods became rhetorical devices as a result of these perceived challenges. The choice of methods itself became dependent on the judicial context. Methods needed to be not only scientifically sound, but also—and perhaps more importantly—convincing for non-experts. In order for analytic methods to become compelling as evidence in the court, they had to be made clear and obvious.

The article is divided into two parts. First, I will give a short overview of the trial reforms in the German states after the revolution of 1848 and explain the challenges for forensic toxicologists that resulted from these reforms by drawing on contemporary textbooks. Second, I will show how these perceived challenges influenced the choice of methods both in theory and in practice. The strategies were informed by two epistemic values which I have called elsewhere redundancy and intuitive comprehensiveness (Carrier 2019). It is the latter of these values which had the greater impact, and which accordingly forms the focus of this section.

## A New Criminal Trial Procedure

After the revolution of 1848, one of the core demands of the liberal revolutionaries was the reform of trial procedure. This old system was a secret and dossier-based procedure, characterized by hard rules of evidence and by the absence of boundaries between prosecutor and judge (Poppen 1984: 60–74; Crawford 1994: 95–100; Watson 2011: 38f.). After 1848, this old procedure was replaced by an oral and public trial by jury where the office of the public prosecutor was distinguished from the office of the judge. Most importantly for the matter of scientific expertise, the strict rules of evidence were replaced by the so-called principle of free consideration of evidence. The strict rules of evidence, originating in Roman-canon law, relied heavily on direct evidence, which, in cases of capital punishment such as poisoning and murder trials, amounted to the confession of the defendant or two testimonies of credible eyewitnesses. All other kinds of evidence—which are commonly referred to as circumstantial evidence—were subordinated to direct evidence and could not justify capital punishment (cf. Donovan 2010: 24, 27f.; Langbein 2006). In this context, the new trial procedure and the principle of free consideration of evidence had the potential to strengthen the position of forensic evidence in the courtroom. Judges and juries were now in a position to decide for themselves which evidence should be seen as credible and how much of this evidence would be needed for a conviction (Donovan 2010: 29f.; Poppen 1984: 221f.). The model of this new criminal trial procedure originated in France where Napoleon had introduced the *Code d’instruction criminelle* in 1808, which had in fact resulted in the reintroduction of inquisitorial aspects after the previous abolishment of the old trial system in 1791 (Donovan 2010: 33–44). Although German nationalists tried to emphasize connections to the English adversarial system, at the core of this new criminal trial procedure was the attempt to combine adversarial and inquisitorial procedures as can be found first in the Napoleonic criminal trial reform (Donovan 2010: 41–43; Poppen 1984: 221f.; Habermas 2008: 166–173; Royer et al. 2016: 404f.). The procedure consisted of a pretrial which was modeled on the inquisitorial model: in this phase, the investigation was led by a judge or a public prosecutor depending on the severity of the crime. This investigation was not open to the public and could not be challenged by the accused party at this stage. The trial itself was constructed with the adversarial system in mind. The two parties, the prosecution and the defendant, then presented their cases to an impartial jury and one or more judges. The jury answered questions on whether they deemed the accused guilty or not, the judge(s) would then set the penalties if the defendant were found guilty. In poisoning cases, chemical and medico-legal expertise was highly relevant during both the pretrial and trial phases to establish whether the suspected crime had actually taken place. The revolution in Germany ultimately was unsuccessful in establishing a German nation-state, however the resulting trial reforms remained in place in most German states and served as the basis for the criminal procedure in Imperial Germany after 1877 when the general code of criminal procedure (*Strafprozessordnung*, StPO) was adopted. Similarly, the French criminal trial procedure was kept intact at its core throughout the nineteenth century.

As already mentioned above, the principle of free consideration of evidence could potentially strengthen forensic evidence in criminal trials by giving judges and juries the right to rely on such circumstantial evidence alone for their sentence. However, the new public trial also threatened to undermine scientific credibility and authority, as some textbook authors argued. They identified two reasons for this risk: first, the risk of public expert disagreement and second, the risk of scientific experts suffering from a rhetorical disadvantage to lawyers. It is the latter of these risks which shall be further considered here.

Toxicology textbook authors feared that medical experts would be confronted with unexpected questions in a public trial to which they might not be able to give a satisfactory answer on the spot. As neither pharmacists nor physicians were trained in public speech in the same way as lawyers were, the authors argued that this put the experts in a criminal trial at a rhetoric disadvantage which could then be used to undermine their credibility publicly and could ultimately lead to the dismissal of scientifically sound evidence. An example of this position can be seen in the work of the physician Friedrich Wilhelm Böcker (1818–1861), who wrote in his textbook *Memoranda der gerichtlichen Medicin* in 1854:

In our times, legal medicine […] gains more and more influence on the civil life through the public trial. Thus, there also is a higher interest in the legal physician; however, his role also becomes considerably more difficult than it was in the secret and written trial. There, the use of books as well as long and quiet contemplation was admissible, whereas in the public and oral trial, the physician must answer promptly to unexpected questions of all parties and must not delay his response. In the public trial, the physician needs greater skill in public speaking and must have a much more thorough overview on the whole field of legal medicine than was the case in the secret trial. In contrast, his good reputation is much more at risk than before, since he is criticized by the audience.^3^ (Böcker 1854: 8)

Not surprisingly, the argument is sometimes tied to the demand for an educational reform in legal medicine. One way to solve the perceived problem was to better educate future experts in public speech so that they would not face rhetorical disadvantages in the courtroom. This argument also fit well with demands to strengthen legal medicine as a special discipline (Schürmayer 1854: 10f.).^4^ However, since in practice the expert in question was not a specially trained legal physician but rather the district medical officer (*Physikus*) who in poisoning cases worked together with a local pharmacist, who also lacked special legal training, this demand was a long-term solution at best. In the short term, forensic toxicology had to rely on different strategies to overcome the problems in the courtroom which were closely linked to the question of the choice of the best analytical methods.

To be sure, it should be stressed again that the strategies that are discussed in the following are directed towards *perceived* risks, not necessarily real ones. There certainly was public expert disagreement. One of the best documented French cases was the Lafarge affair, in which tests for arsenic had to be performed four times by different groups of experts, yielding disparate results until the court was satisfied that a poisoning had in fact taken place (Bertomeu-Sánchez 2006). Another well-documented German non-poisoning case is the murder of the Countess of Görlitz. Here, experts entered into a heated discussion of the possibility of spontaneous combustion of humans (Heilbron 1994). However, in both of these cases, the original expert testimonies were not simply challenged by rhetorically superior attorneys and then arbitrarily rejected by a judge and a jury of lay persons, but rather met with contradicting expertise. Judge and jury certainly had to decide *which* expert to follow in these cases, but the much-feared scenario in which expert testimonies were arbitrarily rejected seems exceedingly rare. I have yet to find a case in France or the German states where the arbitrary rejection of expert testimonies took place.

One must also be wary of the strong distinction between lay persons and experts implied in the authors’ perceived risks of a public trial. As James Donovan (2010: 43f.) has rightly pointed out for the French case, the ability to serve as a member of the jury was restricted to a small social group. Juries could and often would include a high proportion of doctors and other learned men; these individuals likely possessed a basic understanding of the analytical methods in chemistry, thus calling into question the strong distinction between lay-persons and experts. Again, it is not the aim of this article to judge whether the concerns of the forensic toxicologists were actually justified. Given that the authors perceived these problems as possibilities, they acted accordingly to make their testimonies more credible to the jury. It might well be that this was a reaction to a non-existent issue, however in analyzing this reaction, I argue that it is still possible to better understand strategies for claiming authority and gaining credibility in a legal setting.

## Seeing Is Believing: the Rhetorical Use of Methods

Regardless of whether the perceived problems for expert witnesses in the new criminal trial procedure appeared in practice or only in the imagination of the forensic toxicologists, strategies to overcome these problems had to be found. The textbook authors offered three strategies: two of these strategies corresponded to what I have previously called redundancy and intuitive comprehensiveness, the third one attempted to transfer the responsibility of method selection to the state.

### Redundancy

To illustrate redundancy, consider the following quote from the textbook *Die gerichtliche Chemie für Gerichtsaerzte und Juristen* written by the Viennese chemist and physician Franz Schneider (1812–1897) and published in 1852:

[T]he substance has to be examined with *all* characteristic reagents and the chemist must explicitly point out on which reactions he bases his statements on the presence of a substance and in which properties the substance in question differs from all other substances which are related or similar in their effect. Only by performing these steps will his judgment be exhaustive and, at the same time, will it counter those doubts and objections which might be raised by the court or the attorneys.^5^ (Schneider 1852: 6; emphasis in the original)

In this view, the best way to overcome critical questions and the aforementioned risks was not to choose a single method, but rather to use all accessible methods for identifying the substance in question. According to this logic, if one method was not enough to convince the court, the sheer mass of evidence produced by following this strategy surely would succeed. Of course, a stategy of redundancy also diminished the risk of producing false positives, yet this is not how Schneider justifies it here. Instead, he explicitly argues that it provides the best way to face “doubts and objections” from the court; thus it is justified as a defense strategy against lay persons and not (solely) as a way to do better analytic work.

This strategy of employing a great number of redundant methods did not work well in practice. The main reason for this was that there was a tendency in forensic toxicology to strive after and use methods to employ the smallest possible sample size. In poisoning cases, the sample was limited and the court might need to preserve leftovers to ask for second opinions in possible appeals (Carrier 2019: 49).

### The Quarrel About Standard Methods

One possible way to get around the whole question of method selection was the implementation of standard methods set by the state. If the state specified standard methods, the courts could then examine expert testimonies on a purely formal level. This idea was most prominently supported by Carl R. Fresenius (1818–1897) even before the beginning of the reform to the trial procedure. In 1844, he published the article *Ueber die Stellung des Chemikers bei gerichtlich-chemischen Untersuchungen* in which he began by explaining that one of his tasks in this article would be to highlight,

how useful it would be if the state would prescribe well tried and tested methods for the detection of poisons as a standard so that every chemist commissioned with the task of an investigation of a poisoning must follow this standard.^6^ (Fresenius 1844: 276)

Fresenius goes on to explain that every choice that the chemist makes can and will ultimately face harsh criticism in court. He provides an example of a chemist who is commissioned by the court to find arsenic:

If he [the chemist] chooses one of the older methods [to find arsenic] and he cannot detect arsenic, it will be said, “how can one apply such a method when we have much more accurate and better ones?; if one had chosen *Marsh*’s apparatus, one would have found arsenic.” If he chooses *Marsh*’s apparatus and he detects arsenic, the defendant’s attorney will without doubt say: “what can one think about these results, results, that is, which are obtained by a method that can easily be misleading […].” If, finally, the chemist wants to satisfy everybody, and he divides his substance in as many parts as there are methods, examining every part with a different method and cannot find arsenic, it will be said that there is nothing more natural than that result, since every beginner knows that every reaction has a minimum sensitivity […].^7^ (Fresenius 1844: 283f.; emphasis in the original)

Fresenius highlights how the chemist’s choices are restricted and each strategy carries disadvantages. Since the matter came down to a choice, it could always be attacked as possibly not being the best one. Redundancy could not be the answer since then the amount of sample material would simply not be sufficient. The only viable path Fresenius saw for escaping this problem was to let the state make the choice. He drew support for this position from the pharmacopeia, in which directions for how pharmaceuticals should be prepared or checked were written down. Just as the pharmacist was only responsible for acting as prescribed by the pharmacopeia and not for checking each recipe, Fresenius argued, the forensic toxicologist should only be responsible for using the correct standard method and not for deciding on the method itself. The choice of good methods should not be the responsibility of the individual expert but rather of the state (Fresenius 1844: 285; cf. also Reinhardt 2011).

In the textbooks, this plea for officially-set standard methods appears, however it is generally seen as something unrealistic and undesirable. This skepticism also rested on the observation that new methods were found too quickly for the state to react. Every law that would set the catalogue of standard methods was bound to be outdated right after its implementation (Schneider 1852: 5f.). Fresenius had already foreseen this issue in 1844, yet concluded that it was not a decisive problem. Laws, he argued, are always dependent on circumstances and must be changed when the circumstances they rely on change. Of course, it would be the responsibility of the state to adapt the standard methods to the developments in analytic chemistry, but this did not mean, in Fresenius’s view, that the state was unable to set a standard that would be valid for a certain time (Fresenius 1844: 286). He seems to have been less concerned about whether the expert actually used the best method and instead argued that a set of prescribed standard methods would bolster the authority of an expert testimony in courts of law. The methods themselves would then stand above critical questions and would have to be accepted by every layperson involved. However, he did not succeed in convincing his colleagues and there were no legally specified standard methods. To be sure, this did not mean that there was no standardization, but rather that the textbook authors did not want to give up their own right and responsibility to establish and develop these standards in practice.^8^ The Marsh test for arsenic (and its variants) most certainly was a standard method and it was practically used as such, however, it was not legally prescribed but rather set by the community.^9^ To establish such community standards, the textbook authors employed a different strategy in their method selection which was guided by the value of intuitive comprehensibility.

### Intuitive Comprehensiveness, Part I: Showing the Material

The practical usefulness of the next strategy, which adhered to the value which I call intuitive comprehensibility to approximate the meaning of the German word *Anschaulichkeit*, was much greater than both redundancy and the plea for standard methods, which is also why Schneider himself preferred it. In the paragraph directly before the quote about redundancy cited above, he wrote:

Whereas the analytical chemist will choose such detection methods which are either distinguished by their characteristic chemical behavior or allow for the most accurate quantitative analysis, the forensic chemist must aim to present his substances *in such a way that they can easily be recognized and distinguished by everyone*.^10^ (Schneider 1852: 5; my emphasis)

This quote is interesting for two reasons: first, Schneider draws a distinction between analytic chemists and legal chemists that relies on a specific form of method selection, and second, this method selection was oriented towards the abilities of lay persons to recognize certain substances. In Schneider’s view, the ability to find and use these methods convincingly was the defining attribute of professional legal chemists. The discussion of the use of multiple methods cited above was the strategy he recommended only in cases in which such methods were non-existent or for some reason not useable (Schneider 1852: 6). But what did it mean in practice for substances to be in a form which could be easily recognized by everyone? Schneider goes on to give an example:

If, for instance, his [the chemist’s] task is to prove a poisoning by copper or determine the proportion of copper in food products and the like, there is no question that the judge and all laypeople will be much more convinced of the presence of this metal if iron is used to precipitate it in its elementary form—in which it can be easily recognized by everyone due to its copper red color—than if it is dissolved in excess ammonia, which produces the dark blue color by which every chemist detects the presence of copper in qualitative analyses.^11^ (Schneider 1852: 5)

Since even laypeople were familiar with the appearance of copper, seeing a substance which looks like copper would accordingly be convincing enough to establish that the substance in question really was copper. In Schneider’s view, this visual similitude would be more convincing than seeing a solution turn blue and hearing a chemist explain that this change in color demonstrated the presence of copper. In the latter case, the chemist would then have to go on to explain that no other substance would turn the same solution blue and perhaps even demonstrate this process again (Schneider 1852: 6).

This corresponds well with what Burney called “plain matter of fact” (Burney 2012: 78–115). He argued that toxicology gained a special position in poisoning cases precisely because it could reproduce and show the poisonous material:

By enabling experts to present poison in its tangible material form, chemical demonstration held out the promise of disrupting the poisoner’s insidious designs. Through his reproduction of the equivalent of the bloodied dagger in his tubes and retorts, the toxicologist promised to translate this most ephemeral of crimes into a more conventional form of violence. (Burney 2012: 80).

The prime example for putting this idea in practice was the almost exclusive use of the Marsh test for arsenic in poisoning cases. This test was developed by the British chemist James Marsh (1794–1846) and relied on the production of the gas arsenous hydride in a reaction of arsenic, zinc, and sulfuric acid. The gas could then be lit, thus producing a metallic mirror of elemental arsenic on a porcelain or glass surface when held over the flame. If no arsenic is present in the analyzed substance, the gas produced during the test is not arsenous hydride, but hydrogen which does not result in such a mirror when burned (Watson 2006: 192–194; cf. also Burney 2012: 86–101).

The production of the elemental arsenic in the Marsh test is similar to Schneider’s example above. Of course, elemental arsenic does not have the distinctive color of copper, however, showing laypersons an actual metal when claiming that a metal was used as the poison could serve to underline the claim and make it more easily understood and therefore convincing. The Marsh test had the desired attributes of a good analytical test—its high sensitivity, for instance, being an important one^12^—but this usefulness of the Marsh test as a rhetorical tool seems to have contributed to making it the method of choice for identifying arsenic (Carrier 2019: 53). Arsenic was a popular choice among poisoners in the nineteenth century (Watson 2004: 33). However, it was not the only choice and for many of these alternative poisons, toxicologists lacked methods as demonstrative and conclusive as the Marsh test. In the following, I will argue that there was nonetheless a way to use intuitive comprehensiveness and avoid reliance on redundancy in such cases by employing a strategy of explicit comparison.

### Intuitive Comprehensiveness, Part II: Explicit Comparison

Explicit comparison as a rhetorical device does not appear very prominently in the textbooks. To illustrate what I mean by this term, I will give an account of a practical example found in a poisoning case that took place in Springe near Hanover in 1870.

In September 1870, the scribe Fricke in Springe had fallen ill with symptoms of heavy vomiting and diarrhea. His wife, Dorothee Fricke who later was one of the defendants in this case, called the local physician who suspected that the scribe had cholera and tried to treat him for that. However, two days later, on October 2, 1870, Fricke had died. Since there was no sign of a crime at first, Fricke was buried without a postmortem. Only a few days after his death, the journeyman dyer Bernhard Pilz who had lived for nearly a year in Fricke’s house as a boarder wrote his parents that he would either marry the widow Fricke with their consent or never come home again. Pilz’s father refused vehemently, and in November, he came to Springe to bring his son home. However, as the bill of indictment reports, Dorothee Fricke told Pilz’s father that Pilz had killed her husband and that she would denounce him to the police if his father took him away. However, Pilz’s father did not comply and instead took his son with him to the local mayor and the public prosecutor where he himself reported this conversation and let his son make a statement.^13^

This was a rather typical poisoning case in the nineteenth century. As Katherine Watson has shown most poisonings happened within the family.^14^ Out of the 540 cases, she analyzed in 320 (so nearly sixty percent) the main target was part of the family of the poisoner. Wives were one of the biggest groups of poisoners only surpassed by (step-)mothers (Watson 2004: 47).^15^

The poison used in Fricke’s case, however, was not typical. In his statement to the police, Pilz confessed that Fricke’s wife had poisoned her husband using potassium chromate (“chromsaures Kali”^16^) which he had provided. He had obtained the potassium chromate from the dye house where he worked. He was quick to explain that he had never wanted to kill Fricke but he and the widow had started having an affair a month before the scribe’s death. Mrs. Fricke complained about her husband very often. The late Fricke allegedly mistreated his wife when he was drunk—and in fact seems to have been drunk rather often—and was said to be unfaithful to her. As Pilz explained in his confession, she had told him that she could not live with her husband anymore and that she would either run away or poison him and then marry Pilz.^17^ She tried to convince Pilz to provide her with poison from the dye house, but he refused at first. However, a few days later, she found at home a small amount of potassium chromate which Pilz had brought home from work—although he never explained the reason for bringing this home—and which he claims to then have forgotten at home. She gave that to her husband who started to feel ill and when Pilz came home, she told him to bring her more of this substance. Pilz claimed that it was only at this point that he had learned that potassium chromate is actually poisonous and that he started to provide her willingly with more. However, he claimed to never have given Fricke the poison himself.^18^ Mrs. Fricke did not confess to anything and claimed that she had had no affair with Pilz before her husband’s death and that she had never poisoned her husband. If her husband had died by poison, she claimed, Pilz must have been the one giving it to him.^19^

Following Pilz’s confession, Fricke’s body was exhumed, and a postmortem was performed. The protocol of the postmortem, which was performed by the district medical officer and his assistant in the presence of two officers of the court and the prosecutor, reported that no signs of an illness explaining the death of Fricke were found.^20^ However, even if such internal signs had been found, as many textbooks throughout the nineteenth century stress, there was no definitive medical proof of a poisoning which could then be found in symptoms or in a postmortem. Neither symptoms before the death nor appearances on the internal organs, such as inflammation, were specific enough to infer poisoning. All the textbooks stress that symptoms or findings in postmortems could contradict the assumption of poisoning by a certain substance but they could never result in a positive proof (cf. for example Brach 1850: 381f.; Caspar 1860: 408; cf. also Burney 2012: 79f.). The only way to get certain proof that a criminal act had taken place was to gather samples from the body during the postmortem, such as (parts of) internal organs or their contents, which would then be sent from a pharmacist for chemical analysis. In most cases, the court would accompany the samples with clear orders to search for a specific substance or a class of substances based on the results of the preliminary investigation; sometimes questions about the poisonous effects of these substances, that is, what quantity was needed to kill a person, or what the normal symptoms of this poison were also appeared in the orders. Whereas it was important for officers of the court to be present at postmortems, the pharmacist usually would perform the analysis by himself or with an assistant. There was no official witness to oversee his analysis. The textbooks argue that this is mostly a matter of convenience since chemical analyses could take days rather than hours (Brach 1850: 20).

This all was also true in the Fricke case. Samples of different organs and their contents were gathered during the postmortem and sent to the pharmacist Viktor Sertürner (1834–1887) in Hamelin who was commissioned to look for inorganic poisons that could be found in dye houses, especially potassium chromate. There were possible strategies pharmacists could follow when confronted with a completely unknown poison, however, these separation processes took time and material and were avoided when possible. Sertürner systematically excluded the presence of different metals in the sample and finally found chromium. After isolating it, he used two reactions to establish its presence. First, he used silver nitrate which resulted in a red precipitate, and second, he used lead to produce a yellow precipitate. He included both results with his written testimony.^21^

This is exactly the kind of color reaction Schneider frowned upon. Without at least some chemical explanation, there was no reason for laypersons to believe that these red and yellow substances would prove the presence of chromium in the samples: precisely the situation that Schneider wanted to avoid (Schneider 1852: 5). It is possible to understand this behavior as the attempt to adhere to the value of redundancy with limited resources (as I did before, cf. Carrier 2019: 48), which is what Schneider would have recommended in cases where no good method was available to produce the substance in an easily recognizable form. However, it seems to me that redundancy is not sufficient to understand this case of expertise. Sertürner did not use all or even many of the possible methods to detect chromium and he did not use the same methods on different substances to show the different results of this test: he only used two methods, and moreover two very similar methods. In this case, redundancy (in the form of at least more than one method) was combined with another form of intuitive comprehensibility: explicit comparison.

Intuitive comprehensibility always relied on a form of comparison.^22^ When Schneider recommended using forms of substances which are known to everyone, he assumed that everyone had actual experiences seeing the substance in question and thus would be able to recognize it. In the case of Schneider’s recommendation, comparison was implicit; there was no direct reference to the comparison itself. In the Fricke case, however, an explicit comparison was used. In a second letter, Sertürner provided the court with two other precipitates, again red and yellow showing the presence of chromium. This time, he used pure potassium chromium showing that potassium chromium actually behaved in the way he claimed in his first letter. Furthermore, he did not use just any potassium chromate for this demonstration, he used potassium chromate that he obtained from the dye house at which Pilz had worked.^23^ A closer comparison between the alleged poison and his findings in the chemical analysis was hardly possible. Laypersons might have had no prior experience with the chemical behavior of chromium but this demonstration could provide such experience.

Since the protocol of the hearing itself is not to be found in the dossiers, I can only speculate about the effectiveness of this strategy. However, neither the judge nor the jury seemed to have any doubts about the poisoning itself. In the list of questions on which the jury had to deliberate, the question whether the crime itself had taken place was not included. The only questions the jury had to answer were essentially whether Mrs. Fricke had deliberately poisoned her husband, whether Pilz had also deliberately poisoned Mr. Fricke, and whether Pilz had helped Mrs. Fricke by knowingly providing her with poison so that she could poison her husband. The jury decided that Mrs. Fricke had acted alone, although helped by Pilz. She was sentenced to death, but this sentence was later changed to prison for life by a royal pardon. She was released after more than 20 years of prison in 1892 after another royal pardon.^24^ Pilz was sentenced to prison for seven years and released again in 1878.^25^

This example shows another form of intuitive comprehensibility than the one which can be seen in the example Schneider gave in his textbook. Not only methods that produced well-known substances but also this form of explicit comparison can be better understood if seen as a strategy of demonstrating one’s own expertise to laypersons. In the textbooks, however, this kind of demonstration played no role whatsoever. Comparative probes, where the same experiments are done with solutions of known substances, are mentioned to rule out false negatives or false positives but not in this form as a rhetorical device. Usually, the experiments with the original samples and the comparative probes were to be conducted at the same time and under the same conditions to rule out experimental errors or contamination (cf. for example Otto 1884: 12; Schürmayer 1854: 241). In this case, however, it seems that the experiments with the potassium chromate obtained from Pilz’s workplace were done at a later date. There is, after all, no mention of a comparative probe in the extremely detailed description of every single experimental step in the first written testimony^26^; but it was only in the second letter to the court a week after the first written testimony that these comparison probes were mentioned.^27^ Additionally, there was no chemical reason for obtaining the potassium chromate from Pilz’s former workplace. A comparative probe to rule out mistakes could have used any potassium chromate. Understood as a rhetorical device, however, it suggests that not only had potassium chromate been found during the first analysis, but that the detected potassium chromate was furthermore identical to exactly the potassium chromate Pilz had access to.

Comparative probes and the use of explicit comparisons might look similar in practice (because they are, after all, both comparisons), however they are distinguishable by their use for different purposes. In his typology of comparisons, for example, Hartmut von Sass distinguished, among others, between “Vergleichen als Erkenntnisgewinn” and “Vergleiche als erklärende Abkürzungen” (von Sass 2011: 40–42) which might be useful in understanding the difference I see between comparison probes and explicit comparisons. In the context of analytical practice, the comparison is used for knowledge production. The audience of the comparison is the acting scientist himself who gains knowledge about the chemical composition of a probe. In the context of the expert testimony, the audience consists of the judges, juries, attorneys, etc. Here, the comparison acts as an “explanatory shortcut“ and as a means of providing visual aid where the material itself could not be shown. Its function is no longer knowledge production but convincing others of one’s own knowledge. Thus, the comparison becomes a rhetorical device and is no longer just best scientific practice.

The absence of any description of this strategy in the textbooks does not mean that this strategy had never been used before. I have found at least one French case that can be interpreted in the same way. As mentioned above, France had introduced the new criminal trial procedure in 1808, so the French experts had to adopt to a similar situation nearly half a century before the German ones and in this case they seem to have come up with a very similar strategy.

In 1827, Louis Demollière and his wife Thérese Desplaces Demollière were accused of having killed their neighbors with arsenic. The neighbors, a retired gardener named Heurion and his wife, had decided to sell their land before their death under the condition that they would keep the right of residence on their old estate and receive a life annuity in the case of the death of one of them. The Demollière’s agreed and bought the land. In the bill of indictment, it seems to be clear that the Demollière’s did not keep their end of the bargain after Mrs. Heurion had died. Mr. Heurion was said to be malnourished and not satisfied with the treatment by the couple. He asked his nephew for help who inquired with a notary about the possibilities to annul the original contract. Before the contract could be annulled, however, Heurion died after suffering from a very violent and short illness. The symptoms of this illness were the usual vomiting and diarrhea which did not contradict the suspicions of poisoning in the village of Pontachat. Since Heurion got his food from the Demollière’s, who also stood to financially gain from his death, they were the prime suspects in this case.^28^

An official investigation was started and a postmortem was performed on the victim’s body. Again, there was no medical evidence for poisoning, which is why samples were sent to Jean Gallon, the pharmacist in Étampes who was commissioned with the chemical analysis. Whereas in the German case, the pharmacist did his analysis alone, this early nineteenth-century French pharmacist was in fact accompanied by a medical doctor and two officers of the court as official witnesses. White grains suspected to be arsenic were found in the contents of the stomach and first analyzed by the pharmacist. Since the Marsh test was not developed until 1836, Gallot had to use other methods. The details of these methods are not important as he used a total of five different solutions on the white grains. What is important in this context, however, is that he also used the same methods on a solution of pure arsenic in distilled water, showing effects that were similar in this solution.^29^

In this case, the interpretation of this strategy as adherence to redundancy is not far-fetched as the use of five different methods represented quite an effort. Also, the arsenic solution, could serve as a usual comparison probe since the experiments seem to have taken place at the same time. This is all true, but it seems that explicit comparison and thereby adherence to intuitive comprehensibility also played a role here. In the protocol, which was written by the medical doctor and not by the pharmacist himself, the physician wrote that the pharmacist prepared this solution “[p]our donner plus de certitude à nous”.^30^ This suggests an intentional use of the arsenic solution as a rhetorical device—or at least that the physician had understood it as such—and not (only) as a means of doing better analytical work. More importantly, there is no similar comment on the high number of methods used to show arsenic in the protocol, which suggests that this fact in itself might not have been interpreted by the physician as providing more certainty.^31^

After having found arsenic in the body and after having found a pharmacy where Mr. Demollière had bought arsenic two years prior, the case for the court was clear. Mrs. Demollière was sentenced to death since the majority of the jury was convinced that she was the one who had administered the poison. Mr. Demollière was acquitted.^32^

Both the German and the French case show how the value of intuitive comprehensibility could play its role in method selection even if no methods in the sense of Schneider’s recommendation were available. The purpose of methods such as the Marsh test and of the use of an explicit comparison was ultimately the same: the results of the experiments became understandable for lay-persons. The verdict by the expert as to whether or not a poisonous substance had been found in the samples relied on demonstrating experimental results everyone could perceive. To follow this strategy in courts of law meant to make results as obvious as possible; credibility was not only a question of trust in a specific expert but also, and perhaps even more importantly, in specific methods and their evident results.

## Evident Expertise

This article presents and describes different strategies forensic toxicologists adopted or argued in favor of in response to the presence of laypersons in courts of law. For forensic experts, the task of convincing laypersons of the validity of their methods and results had always been a challenge, but this became much more pressing with the introduction of the new Napoleonic criminal trial procedure. This new criminal procedure introduced a jury comprised of laypersons, and at the same time—and perhaps even more importantly—the principle of free consideration of evidence which ultimately gave them the freedom to judge for themselves the value of the expert testimony.

I have argued here that there were three strategies which were discussed on a theoretical level in textbooks or articles. The first strategy represented redundancy, that is to say, it called for using as many methods as possible to verify the results of an analysis. One the one hand, this can be seen as counsel to perform an analysis more thoroughly in order to be more certain of the result which has to be presented at court. However, the presentation of redundancy in textbooks tended to stress that redundancy would produce more compelling evidence for laypersons. Forensic toxicologists saw redundancy as means of warding off potential attacks on individual methods. It was not just about better science; it was about a more convincing display of science.

The second strategy involved bypassing critical questions by transferring the responsibility of method selection from the individual expert to the state. Especially Fresenius believed that this would ensure the expert received better treatment at court. He argued that every choice of methods could be called into question, with the result that the chemist faced heavy criticism at court. The fact that Fresenius argued that it was unimportant whether the state was able to react quickly enough to changes in analytical chemistry shows that he too was more concerned with the authority and the ability to convince laypersons than simply the pursuit of better analytical work.

Both strategies were not put into practice, at least not in the exclusive way in which they are presented here and were argued for in the textbooks. The typically scarce sample material and the imperative to preserve a certain part of the sample for possible future analyses further complicated the pursuit of redundancy. Fresenius’s plea for legally binding standard methods did not fare well among toxicologists, who did not want to give up the freedom of method selection and who argued that state-imposed methods would result in outdated analyses that would ultimately jeopardize the freedom and lives of the defendants.

The third strategy, which complied with the value of intuitive comprehensibility, proved to be the most successful one. I distinguished between two forms of this strategy. In its first form, the strategy entailed using methods which showed the poisonous substance in question in a way that was easily recognizable. If the poison was a copper compound, to reiterate Schneider’s example cited above, the result of the test should look like copper even when other methods may have proved more reliable.

The second form of this strategy involved the explicit comparison between samples. Here, a known composition was used to demonstrate a similar reaction to that of the court provided sample. This strategy, which was discussed in the textbooks in terms of best scientific practice and not as a rhetorical device, provided the laypersons at court with the context they needed to understand the results without any special chemical knowledge. The only premise they had to accept was that two substances which react in the same way in a certain environment are in fact the same substance. Drawing from Sass’s typology of comparisons, I have argued that in the context of expert testimony, the comparison acts as a form of an “explanatory shortcut.” In both the Fricke case and the Demollière case, the expert in question employed such methods to bolster his credibility. In the example cases, an explicit comparison was used together with the strategy of redundancy by employing more than one method to show the similarities between the self-made and the court provided samples. This shows that these two strategies were not mutually exclusive; instead they could complement each other and make the redundant strategy more manageable.

Whereas it can be argued that redundancy also allowed a greater degree of certainty in the results, the same cannot be said for intuitive comprehensibility. Instead, the purpose seems to have been to provide a visual aid in the courtroom and, crucially, to replace the purely redundant strategy and to minimize the use of time and sample material while being perhaps even more convincing. Of course, that does not mean that the methods chosen by the experts who followed this strategy were “bad” or “unreliable.” However, it does reveal that for this strategy to be successful, the credibility of the method used in the context of a criminal trial was in principle independent from its reliability.

The strategy of intuitive comprehensibility became the strategy of choice in response to the problem of laypersons. The goal was to make it so obvious—so evident—that the expert testimony was correct that it was impossible to cast doubt on the results and undermine the authority of the individual expert. Expertise became evident in order to be counted as evidence.

## Acknowledgements

This article is based on talks held in 2019 at the annual meeting of the GWMT in Bonn and at the International Conference on the History of Chemistry in Maastricht. I thank the discussants at both conferences for their valuable questions and insights. Financial support for this research was provided by a dissertation grant from the Evangelisches Studienwerk Villigst and later by the DFG project “Forensic Toxicology in Germany and France during the 19th Century: Methods Development in Judicial Context” (391910812). I would like to thank Julia Engelschalt, Paulina Gennermann, Gina Klein, Carsten Reinhardt, and two anonymous reviewers for their very helpful comments on an earlier version of this paper.

## Footnotes

1 “Die Vergiftung, oder die Hinrichtung eines Menschen durch stille Beybringung irgend eines in geringer Gabe, unter andern versteckten, sehr gefährlichen Mittels, ist eine so leichte Sache, und erfordert von Seiten des Mörders, so wenig Herzhaftigkeit, daß jede niederträchtige, auf Andere gehässige Seele, zu solchem um so mehr aufgelegt seyn muß, je schwerer es ist, den Thäter zu entdecken, und seiner bösen Handlung zu überführen.” All translations by the author.

2 The term “forensic toxicologists” here refers to both authors of theoretical textbooks on this topic. These authors were mostly professors of medicine or chemistry, and practitioners, that is, mostly pharmacists who would be commissioned by the court to perform the analyses in specific cases.

3 “Die gerichtliche Medicin […] gewinnt in unsern Zeiten immer mehr an Einfluss auf das Staatsleben durch das öffentliche Gerichtsverfahren. Sie erhält dadurch ein grösseres Interesse für den Gerichtsarzt; allein die Funktion des Letztern wird dadurch eine bedeutend schwierigere, als bei dem heimlichen und schriftlichen Verfahren. Bei diesem war der Gebrauch der Bücher, ein längeres und ruhigeres Überlegen zulässig, wogegen beim öffentlichen und mündlichen Verfahren dem Arzte unvorhergesehene Fragen von allen Partheien gestellt werden, deren Beantwortung nicht aufgeschoben werden darf, sondern sofort erfolgen muss. Beim öffentlichen Verfahren bedarf der Arzt viel mehr Gewandheit im mündlichen Vortrage, mehr Geistesgegenwart, eine grössere und gründlichere Uebersicht des ganzen Gebietes der gerichtlichen Medicin, als beim heimlichen Verfahren. Dagegen ist sein guter Ruf, weil er der Kritik des Publikums ausgesetzt ist, viel mehr gefährdet als früher.”

4 For examples of how expert communities professionalized and organized themselves in different countries see for example Chauvaud (2000: 17–70) (for France); and Cuenca-Lorente (2017) (for Spain). For the institutionalization of legal medicine in the German states cf. Eulner (1970: 159–179).

5 “[D]ie Substanz [muss] mit allen charakteristischen Reagentien geprüft und [es muss] ausdrücklich hervorgehoben werden, auf welche Reactionen der Chemiker seine Angaben von der Anwesenheit eines Stoffes stützt, und durch welche Eigenschaften sich der Körper von allen übrigen ihm verwandten oder in ihrer Wirkung ähnlichen unterscheidet. Nur dadurch wird sein Gutachten erschöpfend und zugleich jenen Zweifeln und Einwürfen begegnet, welche von Seiten des Gerichtes oder der Rechtsanwälte erhoben werden könnten.”

6 “wie zweckmässig es seyn würde, wenn von Seiten des Staates wohlgeprüfte und bewährt gefundene Methoden zur Ausmittelung der Gifte in der Art als Norm gegeben würden, dass jeder mit der Ausführung der Vergiftungsuntersuchung beauftragte Chemiker dieselben zu befolgen gehalten wäre.”

7 “Wählt er [der Chemiker] eine der älteren Methoden [um Arsen nachzuweisen] und er findet kein Arsen, so wird es heissen, wie kann man eine solche Methode anwenden, da wir im Besitze weit genauerer und besserer sind; hätte man den Marsh’schen Apparat zu Hülfe genommen, so wäre wohl Arsen gefunden worden. Wählt er den Marsh’schen Apparat und er findet Arsen, so wird zweifelsohne des Beklagten Anwalt sagen: was hat man von diesen Resultaten zu halten, von Resultaten, die durch eine Methode gewonnen worden sind, bei der man sich auf alle mögliche Weise täuschen kann […]. Will der Chemiker endlich Allen genügen, theilt er seine Substanzen in so viel Theile, als es Methoden giebt, prüft er jede Parthie nach einer andern und er findet kein Arsen, so wird es heissen, Nichts natürlicher als dies, weiss doch der Anfänger, dass jede Reaction eine Empfindlichkeitsgrenze hat […].”

8 As an example of how standardization by the experts themselves was not seen as problematic, see Friedrich Julius Otto (1809–1870) who wrote: “Mehr noch als in den früheren Ausgaben habe ich mich in der vorliegenden Ausgabe gehütet, Denen, welche nach dem Buche arbeiten, die Wahl unter verschiedenen Methoden zu überlassen. Wer es besser weiss, als ich, bedarf des Buches nicht, wer es nicht besser weiss, den bringt die Wahl in Verlegenheit.” (Otto 1884: XI)

9 For literature on standardization in general cf. Timmermans and Epstein (2010). On the point that scientific practice in general relied on some form of standardization of methods and evaluation practices for “rendering their somewhat ad hoc activities in the laboratory into replicable and reputable public accounts” cf. Brown (1993: quotation on p. 156).

10 “Während der analytische Chemiker insbesondere solche Formbestimmungen der Körper auswählt, welche entweder durch ihr charakteristisches chemisches Verhalten besonders ausgezeichnet sind, oder welche die Menge der Substanz aufs Genaueste zu bestimmen gestatten, muss der Gerichts-Chemiker seinen Substanzen jene Formen vorzüglich zu geben bemüht sein, nach welchen die Körper von Jedermann leicht erkannt und von ähnlichen unterschieden werden können.” This translation has been used before in Carrier (2019: 50).

11 “Handelt es sich z. B. um die Ausmittelung einer Kupfervergiftung oder um den Nachweis eines Kupfergehaltes in Nahrungsmitteln u. dgl., so wird der Richter und mit ihm alle Laien unstreitig von der wirklichen Anwesenheit dieses Metalles viel bestimmter überzeugt werden, wenn dasselbe durch Fällung mittelst Eisen in seinem elementaren Zustande dargestellt wird, wo es durch seine kupferrothe Farbe von dem gemeinsten Mann gekannt ist, als wenn es in überschüssigem Ammoniak aufgelöst, die tief blaue Färbung erzeugt, an welcher der Chemiker bei qualitativen Analysen die Gegenwart des Kupfers entdeckt.”

12 As shown for the example of the Marsh test, a high degree of sensitivity could also be a problem in itself. Orfila had used the Marsh test in his experiments and believed to have found “normal arsenic,” that is an amount of arsenic that would be present naturally in the body. “Normal arsenic” would have been serious trouble for more sensitive tests since there might be no reliable way of distinguishing between natural arsenic and arsenic used for poison. Cf. Bertomeu-Sánchez (2006); Burney (2012: 99–101); Carrier (2019: 45f.).

13 Königliche Kronanwaltschaft zu Celle, Anklageschrift gegen die Witwe Fricke und den Färbergesellen Pilz, 30. März 1871, Nidersächsisches Landesarchiv, Standort Hannover (NLA HA), Hann. 71, C Nr. 127: 2–21, here: 2–5.

14 This of course assumes that the numbers for the English and Welsh cases counted by Watson are comparable to continental Europe and the German states.

15 This should not give the impression that women overall were more likely to kill with poison, just that they were more likely to kill within the family. Overall, the gender ratio of defendants in trials concerning poison was nearly balanced in Watson’s cases (277 women, 263 men) (Watson 2004: 47).

16 Anklageschrift gegen Fricke und Pilz, NLA HA, Hann. 71, C Nr. 127: 5.

17 Anklageschrift gegen Fricke und Pilz, NLA HA, Hann. 71, C Nr. 172: 7f.

18 Anklageschrift gegen Fricke und Pilz, NLA HA, Hann. 71, C Nr. 172: 8f.

19 Anklageschrift gegen Fricke und Pilz, NLA HA, Hann. 71, C Nr. 172: 9.

20 Sektionsprotokoll der exhumierten Leiche Frickes, NLA HA, Hann. 71, C Nr. 125: 39–44.

21 Gutachten im Fall Fricke/Pilz vom 8. Februar 1871, NLA HA, Hann. 71, C Nr. 125: 435–461.

22 Comparison as a practice and as an object of historical and sociological research, rather than as “only” a method has attracted some attention in recent years. Cf. e.g. Grave (2015); Heintz (2016).

23 Gutachten im Fall Fricke/Piz vom 14. Februar 1871, NLA HA, Hann. 71, C Nr. 125: 429–432.

24 Urteil gegen die Witwe Fricke, NLA HA, Hann. 71, C Nr. 126: 38f.; Umwandlung der Todesstrafe für Fricke in eine lebenslängliche Haftstrafe, NLA HA, Hann. 71, C Nr. 126: 58; Begnadigung vom 8. Dezember 1892, NLA HA, Hann. 71, C Nr. 126: 166.

25 Urteil gegen Pilz, NLA HA, Hann. 71, C Nr. 126: 40f.; Entlassungsurkunde vom 7. Juni 1878, NLA HA, Hann. 71, C Nr. 126: 95.

26 Gutachten im Fall Fricke/Pilz vom 8. Februar 1871, NLA HA, Hann. 71, C Nr. 125: 435–461.

27 Gutachten im Fall Fricke/Pilz vom 14. Februar 1871, NLA HA, Hann. 71, C Nr. 125: 432.

28 Acte d’accusation contre Louis Demollière dit Noirot et Thérese Desplaces femmes Demollière, Paris, 21 December 1826, Archives départementales des Yvelines, 2U 178.

29 Rapport du docteur Vimache et du Pharmacien Gallot, Étampes, 17 May 1826, Archives départementales des Yvelines, 2U 178.

30 Rapport du docteur Vimache et du Pharmacien Gallot, Étampes, 17 May 1826, Archives départementales des Yvelines, 2U 178.

31 Rapport du docteur Vimache et du Pharmacien Gallot, Étampes, 17 May 1826, Archives départementales des Yvelines, 2U 178.

32 Archives départementales des Yvelines, 2U 178.
